# Convergent innate immune and regulated cell-death pathways in selected myopathies

**DOI:** 10.3389/fimmu.2026.1871966

**Published:** 2026-07-08

**Authors:** Moe Yamashita, Jaewoo Park, Sehee Park, Hae Ji Kang, Yoon-Seok Chung, Seon Ah Lim, SangJoon Lee

**Affiliations:** 1 Department of Biological Sciences, Ulsan National Institute of Science and Technology (UNIST), Ulsan, Republic of Korea; 2 Department of Biotechnology and Life Science, Tokyo University of Agriculture and Technology, Tokyo, Japan; 3 Division of Acute Viral Disease, Center for Emerging Virus Research, National Institute of Infectious Diseases, National Institute of Health, Cheongju, Republic of Korea; 4 Department of Life Science, Ewha Womans University, Seoul, Republic of Korea; 5 Graduate School of Health Science and Technology, Ulsan National Institute of Science and Technology (UNIST), Ulsan, Republic of Korea

**Keywords:** idiopathic inflammatory myopathies, infectious myopathies, innate immunity, muscular dystrophies, myofiber death, myopathies

## Abstract

Myopathies are a heterogeneous group of skeletal muscle disorders caused by genetic mutations or acquired insults, including inflammation, infection, endocrine imbalance, and toxic exposure. Myopathies affect a substantial number of individuals worldwide and are a significant cause of chronic muscle weakness and disability. Despite diverse etiologies, progressive myofiber injury and degeneration underlie the functional decline across disease subtypes. Accumulating evidence indicates that innate immune activation and regulated myofiber death pathways, including apoptosis, necroptosis, and pyroptosis, contribute to disease progression in selected genetic and acquired myopathies and may represent increasingly actionable therapeutic targets. This review focuses specifically on the interplay between innate immune signaling and the regulation of cell death pathways in skeletal muscle across diverse myopathies. We discuss pattern recognition receptors, inflammasome activation, and cytokine-driven pathways, such as tumor necrosis factor-alpha (TNF-α), type I interferons (IFNs), and interleukin (IL) family signaling, highlighting how these mechanisms amplify inflammation, impair regeneration, and promote myofiber degeneration. To illustrate category-specific mechanisms, we selected representative disorders from each major myopathy group, including Duchenne muscular dystrophy (DMD) as a prototypical DAMP-driven muscular dystrophy, myotonic dystrophy type 1 (DM1) as a model of secondary innate immune activation associated with RNA toxicity-induced cellular stress, dermatomyositis (DM) as a representative inflammatory myopathy, and severe acute respiratory syndrome coronavirus 2 (SARS-CoV-2)-associated myopathy as a clinically relevant model of virus-related muscle involvement and systemic inflammation-associated muscle injury. By integrating evidence across these disease contexts, this review highlights convergent mechanisms in which innate immune dysregulation and regulated myofiber death drive muscle pathology and provide rational targets for mechanism-based therapeutic strategies.

## Classification of myopathies

**Figure 1:**
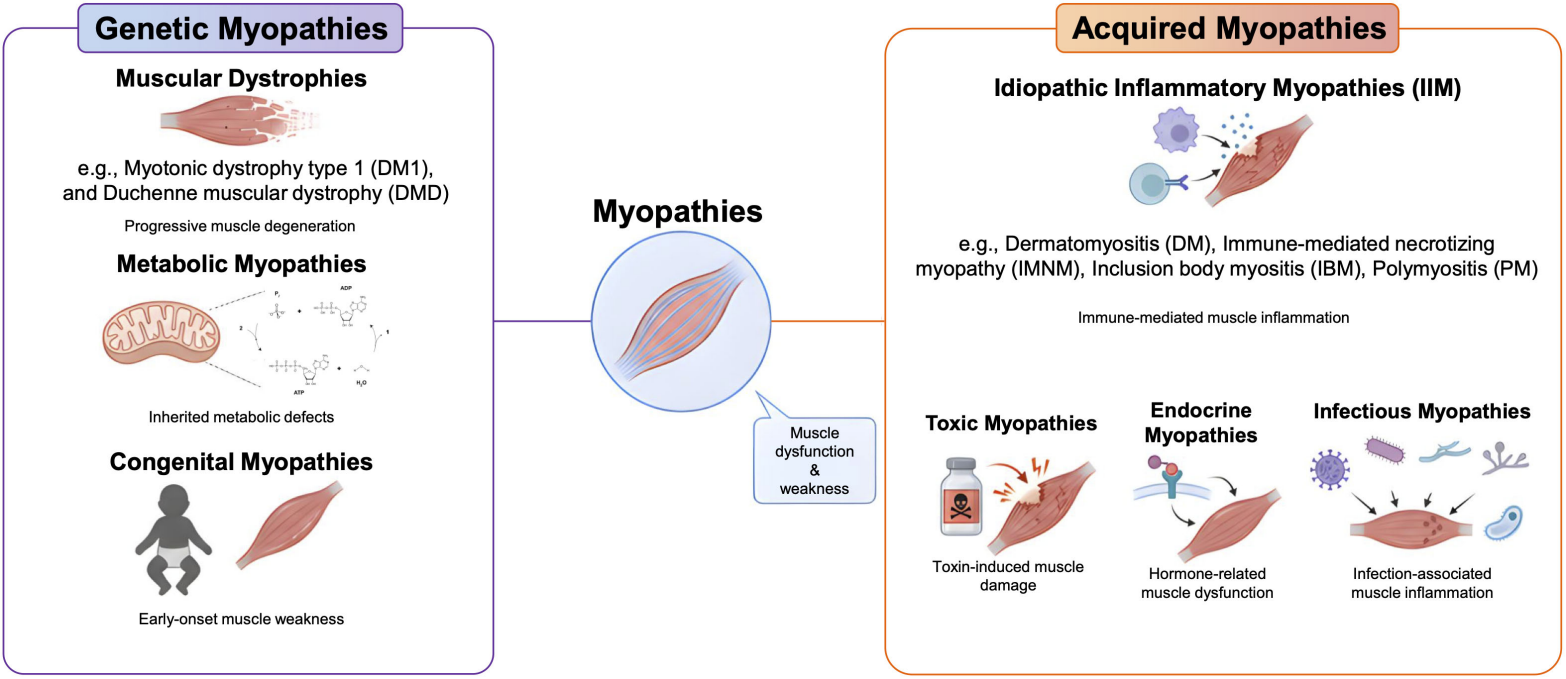
Classification of genetic and acquired myopathies and representative disease subtypes discussed in this review. Myopathies are a heterogeneous group of skeletal muscle disorders characterized by muscle weakness, degeneration, and impaired muscle function. They are broadly classified into acquired and genetic forms. Acquired myopathies include idiopathic inflammatory myopathies (IIMs), toxic myopathies, endocrine myopathies, and infectious myopathies, whereas genetic myopathies include muscular dystrophies, metabolic myopathies, and congenital myopathies. IIMs include dermatomyositis (DM), immune-mediated necrotizing myopathy (IMNM), inclusion body myositis (IBM), and polymyositis (PM). Muscular dystrophies include disorders such as myotonic dystrophy type 1 (DM1), and Duchenne muscular dystrophy (DMD), which are caused by specific genetic mutations and are characterized by progressive muscle degeneration. Infectious myopathies arise secondary to infections caused by pathogens including viruses, bacteria, fungi, and parasites. This review focuses on representative disorders including DM1 and DMD among muscular dystrophies, DM among idiopathic inflammatory myopathies, and SARS-CoV-2-associated myopathy as a representative infection-associated muscle disorder.

Myopathies are a diverse group of disorders that primarily affect the structure, metabolism, or ion channel function of skeletal muscle. Myopathies are usually caused by disruption of muscle tissue integrity, presenting as muscle weakness that interferes with daily activities. They are generally classified into two types: genetic and acquired (**Figure 1**). Genetic myopathies are caused by genetic mutations, whereas acquired myopathies are triggered by metabolic disturbances, inflammation, and imbalances in minerals, electrolytes, and hormone levels. Muscular dystrophies, including myotonic dystrophy type 1 (DM1) and type 2 (DM2), and Duchenne muscular dystrophy (DMD), are representative genetic myopathies. They share clinical features of progressive muscle weakness and a dystrophic appearance on muscle biopsy (1). Metabolic and congenital myopathies also fall within the category of genetic myopathies. Acquired myopathies include idiopathic inflammatory myopathies (IIM), as well as toxic, endocrine, and infectious myopathies. IIM is a heterogeneous group of systemic diseases that lead to muscle weakness, elevated muscle enzymes, inflammation, and extramuscular manifestations (2, 3). The IIM spectrum includes dermatomyositis (DM), inclusion body myositis (IBM), immune-mediated necrotizing myopathy (IMNM), and polymyositis (PM), although PM is currently considered a heterogeneous and increasingly restricted diagnostic category (2, 4–6). Infectious myopathies are secondary myopathies caused by various pathogens, including viruses, bacteria, fungi, and parasites. They arise through direct pathogen invasion or immune-mediated mechanisms, leading to muscle inflammation and weakness, and can overlap with IIM (7).

**Figure 2:**
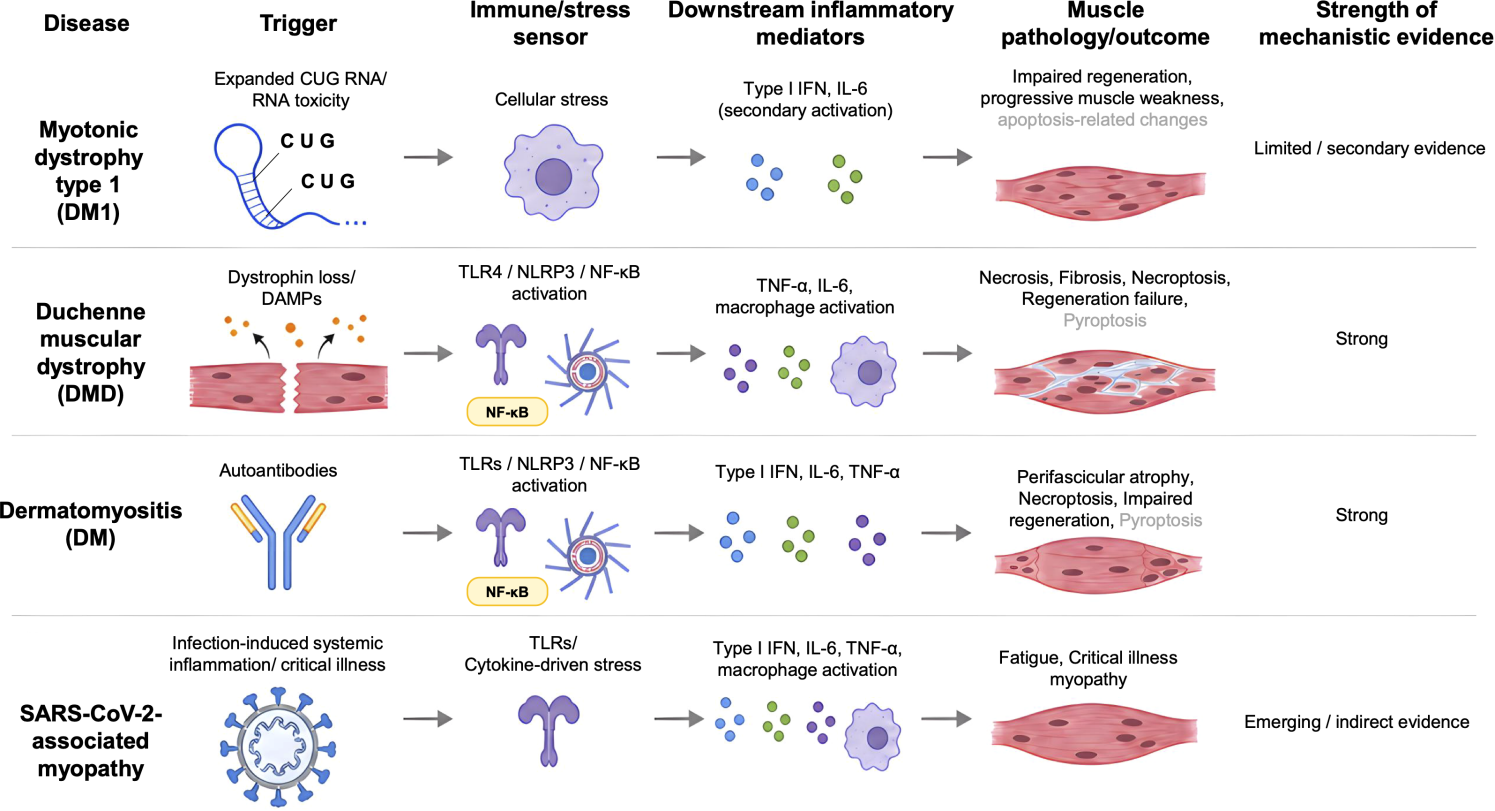
Disease-specific triggers, innate immune/stress sensing pathways, regulated myofiber death mechanisms, downstream inflammatory mediators, muscle pathology, and relative strength of mechanistic evidence across representative myopathies. Distinct pathological triggers engage innate immune-associated pathways through different upstream mechanisms in myopathies. Duchenne muscular dystrophy (DMD) and dermatomyositis (DM) are supported by relatively strong mechanistic evidence linking innate immune activation with regulated myofiber death and muscle pathology, whereas myotonic dystrophy type 1 (DM1) is presented as a model of secondary innate immune activation associated with RNA toxicity-induced cellular stress, and SARS-CoV-2-associated myopathy is shown as a model of systemic inflammation-associated muscle injury with emerging or indirect evidence for regulated myofiber death pathways. The figure highlights both convergent and disease-specific mechanisms underlying inflammatory amplification and muscle degeneration in selected myopathies. The relative strength of mechanistic evidence varies both across diseases and among individual pathways within the same disease context. Gray-colored components indicate mechanisms, inflammatory pathways, or muscle pathology associations currently supported primarily by limited, indirect, secondary, emerging, or context-dependent evidence compared with more established mechanisms shown in standard color.

This review focuses on selected myopathy groups to illustrate how distinct innate immune-associated pathways may drive myofiber injury and degeneration (**Figure 2**).

Among muscular dystrophies, DMD represents a prototypical muscular dystrophy characterized by DAMP-driven inflammation. In contrast, DM1 is included as a model in which innate immune activation is thought to arise secondary to RNA toxicity-induced cellular stress and may contribute to disease progression and myofiber degeneration.

DM is included as a representative inflammatory myopathy characterized by strong activation of innate immune pathways. In addition, SARS-CoV-2-associated myopathy is included as a clinically relevant model of virus-related muscle involvement and systemic inflammation-associated muscle injury, although direct evidence linking it to defined regulated myofiber death pathways remains limited. Together, these disease models provide a framework for understanding how distinct pathological contexts may engage overlapping innate immune-associated pathways and regulated myofiber death mechanisms in myopathy.

## Myofiber death pathway in myopathies

**Figure 3:**
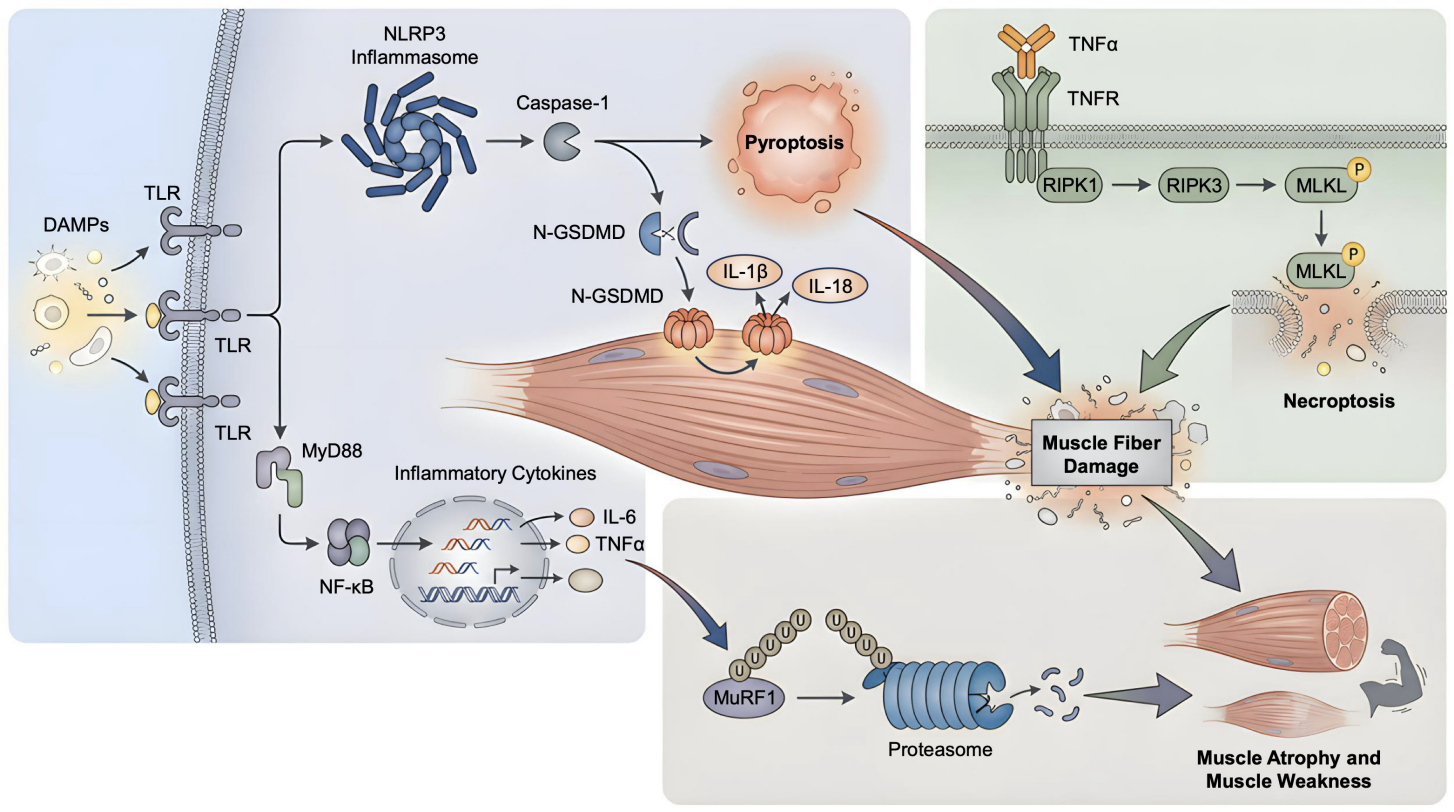
Representative innate immune activation and regulated myofiber death pathways implicated in inflammatory muscle pathology. Disease-associated triggers, including damage-associated molecular patterns (DAMPs) released from injured myofibers, activate Toll-like receptors (TLRs), leading to downstream signaling through myeloid differentiation primary response 88 (MyD88) and nuclear factor kappa B (NF-κB). NF-κB activation promotes transcription of pro-inflammatory cytokines and muscle-specific E3 ubiquitin ligases such as muscle RING finger 1 (MuRF1), resulting in enhanced protein degradation and contributing to muscle atrophy. Concurrently, TLR activation promotes NLRP3 inflammasome assembly, resulting in caspase-1 activation and maturation of interleukin-1β (IL-1β) and interleukin-18 (IL-18), as well as cleavage of gasdermin D (GSDMD), which mediates pore formation and inflammatory cell death through pyroptosis. In parallel, tumor necrosis factor alpha (TNF-α) signaling activates necroptotic pathways via phosphorylation of receptor-interacting protein kinase 3 (RIPK3) and mixed-lineage kinase domain-like protein (MLKL), leading to necroptosis of myofibers. These pathways are most strongly supported in inflammatory and dystrophic muscle diseases such as Duchenne muscular dystrophy (DMD) and dermatomyositis (DM), where they contribute to inflammatory amplification, myofiber injury, impaired regeneration, and muscle dysfunction. The figure summarizes representative inflammatory and regulated cell death pathways implicated in muscle pathology.

In myopathies, multiple regulated cell death pathways contribute to myofiber degeneration (8, 9). Pyroptosis and necroptosis are typically engaged in myofibers under conditions of inflammation and cytokine exposure, as reported in muscular dystrophies such as DMD and in IIM (**Figure 3**) (10–12). These pathways are closely associated with innate immune signaling and inflammatory cell death (9, 13).

In contrast, apoptosis predominantly reflects mitochondrial stress and the intrinsic death pathway and is widely observed in chronic genetic myopathies such as DM1 (14, 15). These pathways often coexist within diseased myofibers, suggesting a coordinated or parallel activation (9, 13, 16).

### Mechanisms of regulated myofiber death

When pathogen-associated molecular patterns (PAMPs) and DAMPs are sensed by pattern-recognition receptors (PRRs), NLRP3 oligomerizes and forms the NLRP3 inflammasome, which activates caspase-1, leading to the secretion of IL-1β and IL-18, which are key mediators of the inflammatory response (17, 18). Caspase-1 and caspase-4/5/11 trigger pyroptosis via cleavage and oligomerization of gasdermin D (GSDMD) (19). Activated GSDMD inserts into the plasma membrane and forms pores, leading to cytoplasmic swelling and the release of intracellular components (20, 21). GSDMD-induced pyroptosis further results in the release of activated IL-1β and IL-18 (22). These events collectively lead to pyroptosis, which is characterized by membrane pore formation, cellular swelling, and release of pro-inflammatory mediators.

Necroptosis occurs in response to various stimuli, including the activation of TNFR1 through binding of TNF-α, as well as IFN signaling mediated by Z-DNA-binding protein 1 (ZBP1) during influenza A virus and SARS-CoV-2 infections (17, 23–26). This process is mediated by mixed-lineage kinase domain-like protein (MLKL). Phosphorylation of receptor-interacting protein kinase 1 (RIPK1) and receptor-interacting protein kinase 3 (RIPK3) triggers MLKL oligomerization. Oligomerized MLKL then translocates to the plasma membrane to form membrane-disrupting pores, inducing membrane depolarization and cell death (27).

Apoptosis is characterized by specific morphological changes in dying cells, including cell shrinkage, nuclear condensation, nuclear fragmentation, and formation of apoptotic bodies (28). Depending on the origin of the death stimulus, apoptosis occurs through either the intrinsic or extrinsic pathway (28). Cytochrome c is released from the mitochondria and binds to apoptotic protease activating factor 1 (APAF1) to form an apoptosome that activates caspase-9 and triggers the caspase cascade in the intrinsic pathway (29). In the extrinsic pathway, FAS, TNFR1, and TNF-related apoptosis-inducing ligand (TRAIL) trigger the recruitment of caspase-8, leading to an apoptotic cascade (30, 31).

## Innate immune mechanisms in myopathies

### Innate immune receptors in skeletal muscle cells

Innate immune signaling plays a fundamental role in the pathogenesis of diverse myopathies (32). Skeletal muscle cells express multiple PRRs, including Toll-like receptors (TLRs), which sense exogenous PAMPs and endogenous DAMPs released during muscle stress and injury (33). Additionally, myofibers express cytokine receptors such as TNF receptors, enabling them to respond directly to inflammatory cytokines and activate intracellular pathways that regulate inflammation, degeneration, and myofiber death (34). These signaling pathways can further engage downstream innate immune effectors, including inflammasome activation, which links danger sensing to pro-inflammatory cytokine maturation and pyroptotic cell death (35).

### Inflammasome activation in myopathies

Inflammasomes are cytosolic multiprotein complexes that act as key sensors of cellular stress and danger signals, leading to activation of caspase-1 and subsequent maturation of pro-inflammatory cytokines such as IL-1β and IL-18, as well as induction of pyroptotic cell death (35). Among these, the NLRP3 inflammasome is the most extensively characterized and is broadly activated in response to diverse stress signals, including DAMPs and metabolic perturbations (36). The NLRP3 inflammasome plays a central role in the development and pathogenesis of inflammation-related skeletal muscle wasting, and contributes to myofiber injury and atrophy in multiple experimental models of muscle degeneration (37–40). In denervation-induced muscle atrophy, NLRP3 inflammasome activation induces pyroptotic cell death and upregulates the muscle-specific E3 ubiquitin ligases muscle RING finger 1 (MuRF1) and atrogin-1, key mediators of proteasomal protein degradation and muscle wasting (40). The increased expression of these genes shifts muscle protein homeostasis from synthesis to degradation, resulting in the loss of muscle mass (41).

Genetic ablation of NLRP3 in mice results in lower expression levels of pro-caspase-1 and pro-IL-1β mRNA and pyroptosis-associated proteins in muscle tissue, and attenuates muscle atrophy (40, 42). In addition, NLRP3 deficiency suppresses age-related muscle loss, limits the reduction in glycolytic myofiber size, and improves muscle performance *in vivo*. Consistent with these findings, genetic inhibition of NLRP3, including shRNA-mediated knockdown, markedly reduces pyroptosis and atrophic responses in C2C12 myotubes (40).

### TNF-α signaling activation in myopathies

TNF-α is a central pro-inflammatory cytokine in myopathic conditions and is primarily produced by activated macrophages and T cells within inflamed muscle tissue. Binding of TNF-α to its receptors, particularly TNFR1, activates downstream signaling pathways including nuclear factor-κB (NF-κB) (43). NF-κB is a key transcription factor that triggers the expression of pro-inflammatory cytokines and increases the expression of ubiquitin-proteasome system proteins such as MuRF1, which lead to muscle wasting (44). Sustained NF-κB activation further contributes to muscle degeneration by inhibiting myogenic differentiation through suppression of MyoD expression, impairing muscle regeneration (45).

In addition to its role in muscle atrophy, TNF-α signaling directly triggers myofiber death through multiple regulated cell death pathways. In C2C12 myotubes, TNF-α stimulation has been shown to induce caspase-8– and caspase-3–dependent cleavage of gasdermin E (GSDME), leading to pyroptosis-like inflammatory cell death via the TNFR1-associated complex IIb pathway (46). Furthermore, stimulation of C2C12 myoblasts with TNF- α and the pan-caspase inhibitor Z-VAD results in the overactivation of necroptosis and a significant increase in necrotic cell death (10).

Members of the TNF superfamily beyond TNF-α also contribute to muscle wasting. TNF-like weak inducer of apoptosis (TWEAK) is a small pleiotropic cytokine belonging to the TNF superfamily that contributes to muscle atrophy (47). TWEAK signaling activates NF-κB and upregulates MuRF1 expression, thereby enhancing proteasomal degradation and muscle loss (47).

### TLR signaling activation in myopathies

Skeletal muscle cells express multiple Toll-like receptors (TLRs) that sense microbial products, viruses, nucleic acids, and DAMPs, leading to inflammation (33, 48). Several TLRs, including TLR2, TLR3, TLR4, and TLR7, are expressed in skeletal muscle cells, whereas TLR9 appears to be context-dependent and may vary depending on the cell type and conditions (33, 49). TLR2 and TLR4 are extracellular receptors that recognize many DAMPs, whereas TLR3, TLR7, and TLR9 are intracellular receptors that detect RNA and DNA in endosomal compartments (48).

Activation of TLR signaling in myopathies is largely driven by endogenous DAMPs derived from damaged or stressed muscle fibers, including high-mobility group box 1 (HMGB1), and mitochondrial DNA (33). Engagement of TLRs in muscle cells triggers downstream signaling through adaptor molecules such as myeloid differentiation primary response 88 (MyD88), leading to activation of NF-κB and IFN regulatory factor pathways, thereby promoting inflammatory gene expression, muscle atrophy, and myofiber dysfunction (33, 48). Consistent with this mechanism, the TLR2/TLR4/NF-κB p65 signaling pathway is activated by serum amyloid A1 (SAA1), resulting in myocyte atrophy (50).

### Type I IFN signaling activation in myopathies

Type I IFN binds to the interferon-α/β receptor (IFNAR), which activates the JAK–STAT pathway and induces interferon-stimulated genes (ISGs), mediating antiviral and pro-inflammatory responses (51). Activation of type I IFN signaling in myopathies is frequently driven by nucleic acid–sensing pathways, including TLRs and cytosolic PRRs, which promote sustained ISG expression within myofibers (52). Sustained type I IFN signaling has been implicated not only in inflammatory amplification but also in direct skeletal muscle dysfunction. In a bioengineered pediatric skeletal muscle model, exposure of healthy muscle tissue to type I IFNs, particularly IFN-β, induced IFN-responsive and pro-inflammatory gene expression, oxidative phosphorylation defects, impaired myogenesis, and contractile dysfunction (53).

### IL receptor signaling activation in myopathies

Cytokines of the IL family play a central role in regulating inflammatory responses in skeletal muscle. Members of the IL-1 family, including IL-1β, IL-18, and IL-33, signal through IL-1 family receptors to activate downstream inflammatory pathways that contribute to immune-mediated muscle pathology.

Among IL family cytokines, IL-6 has been strongly implicated in muscle weakness and myopathic processes (54). IL-6 signaling activates the JAK–STAT pathway, particularly signal transducer and activator of transcription 3 (STAT3) and inhibits myogenic differentiation of C2C12 myoblasts (55). In addition to its effects on differentiation, IL-6–STAT3 signaling contributes to the pathogenesis of myositis by promoting inflammatory gene expression and impairing muscle regeneration, thereby exacerbating muscle damage and functional decline (54). Consistently, IL-6–deficient mice develop myositis with a lower incidence and reduced severity in the C-protein-induced myositis (CIM) model (56), whereas blockade of IL-6 receptor signaling completely prevents muscle atrophy in IL-6 transgenic mice (57).

IL-6 production in the skeletal muscle cells is tightly regulated by inflammatory cytokine networks. IL-6 production is induced by a combined stimulation with TNF-α and IL-1β in normal myoblasts (58). In addition, IL-17 and IL-1β induce IL-6 in normal skeletal myoblasts, and their combination further increases IL-6 production (59).

In addition to IL-6, IL-15 has emerged as an inflammation-responsive cytokine in skeletal muscle, with its expression increasing dose-dependently in normal skeletal myoblasts following stimulation with IFN-γ, IL-1α, IL-1β, and TNF-α (60).

### Interaction between innate immune activation and muscle pathology

Innate immune activation in myopathies occurs within a complex tissue environment shaped by regeneration, fibrosis, mitochondrial stress, vascular injury, macrophage phenotypic transitions, and chronic denervation-like changes (32). Persistent inflammatory signaling not only promotes myofiber death but also interacts with satellite-cell–mediated regeneration and extracellular matrix remodeling, thereby contributing to fibrotic and adipogenic replacement of muscle tissue (32). Macrophage phenotypes dynamically shift during muscle injury and regeneration, thereby influencing the balance between inflammatory injury, tissue remodeling, and repair (61). In addition, mitochondrial dysfunction and neuromuscular instability may further amplify DAMP release and sustain chronic innate immune activation (62). These interactions suggest that innate immune pathways participate not only in inflammatory injury but also in broader processes governing muscle degeneration, regeneration, and tissue remodeling in myopathies.

## Myofiber death and innate immune activation in myopathies

Many myopathies share common pathological mechanisms involving innate immune signaling and regulated cell death pathways despite their diverse etiologies. Increasing evidence suggests that these processes contribute to myofiber degeneration and disease progression across both genetic and acquired myopathies. Skeletal muscle fibers can respond to endogenous danger signals through pattern-recognition receptors, leading to activation of inflammatory pathways including inflammasome, TNF-α, type I IFN, and IL-mediated signaling. These pathways may contribute to myofiber dysfunction, impaired regeneration, and myofiber death.

In parallel, regulated cell death pathways such as necroptosis, pyroptosis, and apoptosis have emerged as important contributors to muscle pathology. The relative contribution and pathological significance of these pathways vary among disease contexts. While some myopathies are characterized by prominent innate immune activation and regulated myofiber death, others exhibit more indirect, secondary, or context-dependent involvement of these mechanisms. The following sections discuss how these pathways contribute to representative genetic and acquired myopathies.

### Genetic myopathies

#### 1. Myotonic dystrophy type 1 (DM1)

DM1 is the most common adult-onset muscular dystrophy and is caused by an expansion of a CTG repeat in the 3’ untranslated region (UTR) of the DMPK gene (63). Mutant *DMPK* transcripts aggregate into nuclear foci and sequester RNA-binding proteins, resulting in widespread pathological consequences, including muscle weakness, myotonia, cardiac abnormalities, cataracts, and cognitive impairments (64). Congenital myotonic dystrophy (CDM) is an early, severe form of DM1 characterized by extremely large CTG expansions that are frequently associated with abnormal CpG methylation in *DMPK* (65).

In DM1, apoptosis and autophagy are activated; however, extensive myofiber loss due to acute apoptosis does not appear to be a dominant pathological feature. Instead, myofiber degeneration is thought to result from the progressive failure of cellular maintenance and survival mechanisms under chronic stress, including mitochondrial dysfunction and DNA damage. In a *Drosophila* model of DM1, excessive activation of apoptosis and autophagy induced by expanded CUG-repeat RNA directly contributes to impaired muscle maintenance and progressive muscle wasting (14). Consistent with these findings, intrinsic apoptotic pathways are activated in human DM1 myotubes, as evidenced by cytochrome *c* release, caspase-9 and caspase-3 activation, and DNA fragmentation (15). Concomitantly, altered expression of autophagy-related markers, including LC3 and p62, is observed in these cells (15).

Importantly, expanded CUG repeat RNA disrupts cellular homeostasis and induces chronic cellular stress, including mitochondrial dysfunction and metabolic impairment, which are closely associated with premature cellular senescence in myogenic and muscle stem cells (66–68). These cumulative defects in cellular maintenance and regeneration are thought to contribute to progressive myofiber degeneration in DM1. Although RNA toxicity is considered the primary pathogenic mechanism underlying myofiber degeneration in DM1, accumulating evidence suggests that cellular stress-associated innate immune activation may secondarily modulate disease progression through cytokine- and IFN-related pathways, including TNF superfamily, type I IFN, and IL-6 signaling (69).

The expression of fibroblast growth factor–inducible 14 (Fn14), the receptor for TNF-like weak inducer of apoptosis (TWEAK) correlates with the severity in DM1 mouse models and in muscle tissues from patients with DM1 (70). In preclinical DM1 models, genetic deletion of Fn14 or antibody-mediated inhibition of TWEAK/Fn14 signaling attenuates inflammatory and dystrophic changes and is associated with partial improvement in muscle function (70).

In addition, upregulation of type I IFN-related genes is detected in cataract tissues of patients with DM1 (71). Expanded CUG repeat RNA can adopt a double-stranded RNA-like structure, which may act as a ligand for innate immune RNA sensors, thereby triggering the activation of type I IFN signaling. IFN-stimulated gene (ISG) signatures are consistently upregulated in the blood and fibroblasts of patients with DM1 (72). Moreover, type I IFN activation contributes to impaired myogenic differentiation and severe muscle manifestations in a subset of patients with CDM (73).

Furthermore, inflammatory cytokine signaling pathways involving IL-6 are dysregulated in DM1 cells. The IL-6 myokine signaling pathway is upregulated in CDM, and IL-6 expression levels significantly correlate with muscle immaturity, a defining pathological feature of CDM (74). In addition, serum IL-6 levels in patients with DM1 positively correlate with muscle weakness and impaired functional capacity (68).

Overall, current evidence supports a secondary and context-dependent contribution of innate immune activation to DM1 pathology, whereas RNA toxicity remains the primary pathogenic driver (**Figure 2**).

#### 2. Duchenne muscular dystrophy (DMD)

DMD is the most common form of childhood-onset muscular dystrophy and is caused by mutations in the *dystrophin* gene. Dystrophin deficiency causes sarcolemmal instability, calcium overload, and the release of damage-associated molecular patterns (DAMPs), which together promote innate immune activation and progressive muscle pathology in DMD (**Figure 2**). The disease affects approximately one in every 3,500–5,000 male births worldwide and is characterized by progressive muscle degeneration, chronic inflammation, and replacement of muscle tissue with fibrotic and adipose tissue (1).

In DMD, myofiber death has long been recognized to occur predominantly through necrosis, driven by sarcolemmal instability, calcium overload, and mechanical stress. In addition to this primary necrotic process, accumulating evidence indicates that regulated cell death pathways are secondarily activated and may further amplify muscle degeneration, inflammation, and fibrosis during disease progression, with necroptosis representing the best-characterized pathway in DMD.

Necroptosis contributes to myofiber death in both mdx mice and patients with DMD, as genetic ablation of RIPK3 in mdx mice reduces myofiber degeneration, fibrosis, and inflammatory infiltration, while improves muscle function (11). RIPK3 expression in the skeletal muscles of dogs with GRMD (a canine model of DMD) is positively correlated with the extent of myonecrosis (9).

In addition to necroptosis, components of the inflammasome and the pyroptotic machinery are upregulated in dystrophic muscles. In a rodent model of DMD, increased expression of gasdermin D, apoptosis-associated speck-like protein containing a CARD (ASC), and cleavage of inflammatory cytokines is observed in skeletal muscles, suggesting activation of inflammasome-related pathways (12).

Apoptotic signaling has also been detected in DMD, although apoptotic nuclei are very rare in normal muscle (75).

Collectively, these findings suggest that while sarcolemmal fragility- and calcium-mediated necrosis remain the primary mechanism of myofiber loss in DMD, necroptosis may act as a major secondary disease-modifying pathway, whereas inflammasome-associated pyroptotic signaling and apoptosis may further contribute to inflammatory amplification and muscle pathology.

Innate immune activation is another prominent feature of DMD and is closely linked to ongoing muscle damage and myofiber death. TNF-α expression was observed in DMD myofibers, but was rare or absent in controls (76). Consistently, TNF-α expression is higher in DMD muscles than in controls and increases significantly with age (77).

In dystrophic muscles, DAMPs contribute to sustained innate immune signaling. In *mdx* mice, the expression of TLR4 and its endogenous ligand HMGB1 is elevated (78). Genetic ablation of *TLR4* in *mdx* mice reduces inflammation, limits macrophage accumulation in dystrophic muscles, promotes anti-inflammatory macrophage polarization, decreases fibrosis, and improves muscle force generation (78). Similarly, treatment with glycyrrhizin, an inhibitor of the endogenous TLR4 ligand, HMGB1, improves dystrophic pathology in *mdx* mice (78). In addition, the endogenous TLR2/4 ligand heat shock protein 70 (HSP70) is significantly upregulated in the serum of patients with DMD compared to age-matched controls (79).

Pro-inflammatory cytokine signaling, particularly involving IL-6, contributes to chronic inflammatory remodeling and disease progression in DMD. IL-6 expression is increased in the skeletal muscles of *mdx* mice and in the serum of patients with DMD compared to controls (80). In dystrophin/utrophin double-knockout mice, treatment with the anti-IL-6 receptor antibody MR16–1 inhibits the IL-6 signaling pathway, improves muscle fiber diameter and regeneration, and reduces fibrosis (81). Genetic ablation of the endogenous IL-6 receptor or pharmacological inhibition of IL-6 signaling confers resistance to muscle degeneration by alleviating the morphological and functional consequences of dystrophin deficiency and promoting an anti-inflammatory muscle environment that enhances repair (80). Moreover, treatment with growth hormone secretagogues (GHSs) decreases IL-6 levels and modulates disease progression in *mdx* mice (82).

### Acquired myopathies

#### 1. Dermatomyositis (DM)

DM is an acquired inflammatory myopathy that presents with an acute or insidious onset of progressive proximal muscle weakness, often accompanied or preceded by characteristic cutaneous manifestations (83–85). Serum creatine kinase (CK) levels are frequently elevated in patients with DM. Myositis-specific antibodies, including anti-melanoma differentiation-associated protein 5 (MDA-5), anti-nuclear matrix protein 2 (NXP-2), anti-nucleosome remodeling deacetylase complex protein Mi-2 (Mi-2), anti-transcription intermediary factor 1 gamma (TIF-1γ), and anti-SUMO-activating enzyme subunit 1 (SAE-1) antibodies, are detected in approximately 60% of patients and are associated with distinct clinical phenotypes (86).

DM is characterized by a distinctive pathological framework involving complement-mediated microangiopathy, perifascicular muscle injury, strong type I IFN activation, and myositis-specific autoantibody-associated immune responses (87). In DM muscle, vascular injury and immune-mediated stress promote perifascicular pathology and the release of DAMPs, which further amplify innate immune sensing pathways (88). These processes are accompanied by robust expression of IFN-inducible proteins such as myxovirus resistance protein A (MxA), sustained inflammatory signaling, impaired regeneration, and activation of regulated myofiber death pathways (88). Among the myopathies discussed in this review, DM currently represents one of the strongest disease models linking innate immune activation to regulated myofiber death and muscle pathology (**Figure 2**).

In DM, multiple regulated cell death pathways are activated in affected muscle fibers. Increasing evidence indicates that myofibers undergo necroptosis in patients with DM (13). Expression levels of RIPK3, mixed-lineage kinase domain-like protein (MLKL), and their phosphorylated forms are significantly increased in muscle tissues from patients with DM compared to healthy controls, and their expression correlates with the severity of muscle damage (10).

In addition to necroptosis, inflammasome-associated pathways are activated in DM muscle. The expression of NLRP3 inflammasome components, including NLRP3, caspase-1, IL-1β, and IL-18, is upregulated in muscle tissues from patients with DM (89, 90). Furthermore, increased expression of pyroptosis-related proteins and mitochondrial apoptosis–associated molecules, including gasdermin E (GSDME), caspase-3, BAX, and cytochrome *c*, has been reported in DM muscle samples, suggesting concurrent activation of inflammatory and apoptotic signaling pathways (16). Collectively, these findings indicate that myofiber injury in DM is mediated by overlapping regulated cell death mechanisms, rather than by a single dominant pathway.

Innate immune activation is a central pathological feature of DM and is driven by persistent tissue damage and immune-mediated stress within skeletal muscle. In DM, injured myofibers exhibit increased expression of DAMPs, which serve as upstream triggers for innate immune sensing and inflammatory amplification.

Among these DAMPs, HMGB1 is markedly upregulated in DM muscle tissues and particularly enriched in myofibers that exhibit overactivated necroptosis (10, 90). HMGB1 functions as an endogenous ligand for TLR2 and TLR4, thereby promoting innate immune activation in DM muscle. The expression of TLR2 and TLR4 is consistently increased in DM muscle fibers (91). In addition, nucleic acid–sensing TLRs are activated in DM, and TLR3 and TLR7 expression is elevated in DM muscle tissues (92). TLR3 and TLR7 proteins are detected in inflammatory infiltrates within muscle tissue from patients with DM, but are absent in healthy control muscle (93). Furthermore, TLR7/8 activation by RNA-containing immune complexes formed by autoantibodies from patients with DM stimulates healthy PBMCs, inducing activation of type I IFN signaling, IL-6 production, and NF-κB–mediated inflammatory signaling (94).

Downstream of innate immune sensing, NLRP3 inflammasome-associated signaling is increased in DM muscle tissues and serum, with upregulation of NLRP3, caspase-1, IL-1β, and IL-18 activation (89, 90, 95). Inflammasome activation has also been linked to inflammatory cell death pathways in DM muscle (90). In parallel, the cytosolic DNA sensor absent in melanoma 2 (AIM2) is implicated in DM progression (96). AIM2 regulates the expression of pyrin and ZBP1, promoting inflammatory cell death and apoptosis during host defense responses to infection (97). Although direct evidence in DM is still limited, these findings suggest that AIM2-dependent pathways may contribute to inflammatory cell death and tissue damage in the DM muscle.

A central hallmark of DM is robust activation of type I IFN signaling. Robust expression of type I IFN–inducible genes, including MxA, is consistently observed in DM muscle fibers (52). Sarcoplasmic MxA expression is proposed as a highly sensitive diagnostic marker of DM (98). Notably, MxA has also been reported to function as an inflammasome sensor during influenza A virus infection in human respiratory epithelial cells, highlighting potential link between IFN-inducible proteins and inflammasome activation across different cellular contexts (99). Disease activity, as assessed by global visual analog scale (VAS) scores, correlates with both type I IFN gene signatures and chemokine expression profiles, and longitudinal changes in IFN activity parallel clinical disease severity (100, 101). Importantly, type I IFN signaling impairs the regenerative capacity of muscle stem cells derived from patients with DM, thereby contributing to persistent muscle weakness (102). Furthermore, recent ex vivo studies have provided additional evidence supporting a direct pathogenic role for type I IFN signaling in muscle dysfunction. Exposure of healthy skeletal muscle to serum from patients with DM induced muscle weakness, whereas blockade of IFNAR1 signaling or downstream JAK-STAT pathways prevented these effects, suggesting that circulating type I IFN–associated factors directly contribute to impaired muscle contractility (103).

These innate immune pathways converge to promote a pro-inflammatory cytokine milieu that exacerbates muscle pathology. Expression of TNF-α is increased in DM muscle tissues and correlates with disease severity (95, 100, 104).

Serum levels of IL-6 and IL-8 are elevated in patients with DM and are associated with disease activity (100, 101, 104). In addition, IL-1β and IL-18 are upregulated in DM serum and muscle fibers (89, 95). IL-17 expression is detected in muscle from patients with DM (59), while IL-15 and IL-15Rα–positive cells are significantly increased; higher IL-15 levels correlate with poorer recovery of muscle function (105).

Collectively, these findings indicate that DM is characterized by sustained activation of innate immune sensing and amplification pathways associated with type I IFN signaling, complement-mediated vascular injury, perifascicular pathology, inflammatory cell death, impaired regeneration, and persistent muscle dysfunction.

#### Virus-induced myopathies

Virus-induced myopathies are acquired muscle disorders that arise secondary to viral infections and are distinct from inherited or autoimmune myopathies. Viruses associated with infectious myopathies include the human immunodeficiency virus (HIV), coxsackieviruses A and B, influenza A and B viruses, human T-cell leukemia virus type 1 (HTLV-1), and SARS-CoV-2 (7).

SARS-CoV-2 primarily infects respiratory epithelial cells. However, the infection is associated with a broad spectrum of acquired neuromuscular complications, collectively referred to as SARS-CoV-2–associated myopathies. These conditions represent secondary muscle involvement following viral infection and fundamentally differ from inherited or autoimmune myopathies.

Musculoskeletal symptoms are not restricted to severe disease. Even individuals with mild-to-moderate COVID-19 frequently report myalgia, fatigue, and persistent muscle weakness, which may continue as part of the post-acute sequelae of SARS-CoV-2 infection (106). These manifestations are thought to reflect systemic immune activation and inflammatory responses that disrupt muscle homeostasis rather than direct viral cytotoxicity (107, 108).

In severe cases of coronavirus disease 2019 (COVID-19), patients may develop inflammatory myositis (107, 109). In addition, critical illness polyneuropathy and myopathy (CIP/CIM) occur frequently in patients with severe COVID-19 requiring intensive care support (110). Angiotensin-converting enzyme 2 (ACE2), the cellular receptor for SARS-CoV-2, is expressed on the membranes of skeletal muscle fibers in critically ill patients regardless of SARS-CoV-2 infection status (111). While one study reported low or undetectable viral loads in most skeletal muscle biopsies from patients who died from COVID-19 (107), another study detected SARS-CoV-2 viral RNA within myofibers in a subset of critically ill patients (111), suggesting that direct muscle infection may occur under specific pathological conditions.

Direct evidence of the activation of defined myofiber death pathways in SARS-CoV-2–associated myopathy remains limited. Histopathological analyses of skeletal muscle from critically ill patients with COVID-19 have predominantly revealed muscle fiber atrophy, degeneration, and fibrosis. However, definitive features indicative of specific regulated cell death pathways have not been consistently demonstrated. Indeed, the activation of fibrotic signaling pathways is observed in the skeletal muscles of critically ill patients with SARS-CoV-2 infection compared with controls (111).

At present, muscle pathology in SARS-CoV-2–associated myopathy appears to be driven largely by indirect mechanisms, including systemic inflammation, hypoxia, metabolic stress, immobilization, and critical illness, rather than by robust activation of necroptosis, pyroptosis, or apoptosis within myofibers.

Innate immune activation, particularly type I IFN signaling and systemic inflammatory responses, plays an important role in muscle pathology associated with SARS-CoV-2 infection. Capillary expression of the IFN-stimulated protein MxA is detected in the skeletal muscle of some patients who died from COVID-19, indicating the activation of IFN-mediated innate immune pathways within the muscle tissue (107). Consistent with this observation, elevated circulating levels of MxA are associated with increased disease severity in patients with SARS-CoV-2 infection, further supporting the involvement of systemic IFN activation in COVID-19–associated muscle pathology (112).

In addition to IFN signaling, structural and ultrastructural abnormalities in the skeletal muscle of critically ill patients with COVID-19 reflect immune-mediated and metabolic stress. Muscle biopsies from intensive care unit (ICU) patients demonstrate myopathic changes consistent with critical illness myopathy, accompanied by variable inflammatory infiltration, autophagic vacuoles, mitochondrial abnormalities, and occasional SARS-CoV-2–immunostaining–positive fibers or granules (113). These findings suggest that muscle dysfunction arises, in part, from inflammation-associated cellular stress and impaired metabolic homeostasis.

Systemic inflammatory cytokine responses contribute to muscle involvement in severe COVID-19. Patients with severe disease exhibit elevated circulating levels of pro-inflammatory cytokines, including IL-6, TNF-α, and IL-1β (114), which are known to promote muscle catabolism, impair regeneration, and exacerbate inflammatory signaling. Furthermore, recent studies have shown that in neutrophils, inflammation-related genes such as S100A9, MMP9, and FPR2 are upregulated and contribute to degranulation and extracellular trap formation, thereby shaping the systemic inflammatory environment, which may indirectly affect muscle homeostasis (115). Together, these findings indicate that SARS-CoV-2–associated myopathy is driven primarily by IFN-mediated innate immune activation and systemic inflammatory responses, rather than by the direct activation of defined myofiber death pathways.

In rare cases, immune-mediated inflammatory myopathies have been reported following mRNA-based SARS-CoV-2 vaccination (116–119). These conditions are thought to arise from transient immune activation rather than direct viral effects. mRNA SARS-CoV-2 vaccination induces type I IFN responses in the peripheral blood mononuclear cells of healthy individuals (120), consistent with previous studies demonstrating that mRNA vaccines broadly activate innate immune pathways, particularly type I IFN signaling (121). Moreover, MxA expression is observed in blood vessels and muscle fibers in cases of post-vaccination inflammatory myopathy (119).

These findings suggest that excessive or dysregulated IFN signaling may underlie muscle inflammation in susceptible individuals following vaccination, providing mechanistic parallels to the IFN-driven muscle pathology observed during severe SARS-CoV-2 infection.

Collectively, current evidence primarily supports an indirect and systemic inflammatory contribution to SARS-CoV-2-associated muscle pathology, whereas direct evidence linking defined regulated myofiber death pathways to disease progression remains limited (**Figure 2**).

### Therapeutics of myopathies

Therapeutic strategies for myopathies have traditionally focused on supportive care and broad immunosuppression, including corticosteroids and general anti-inflammatory agents, particularly in inflammatory myopathies (122). In genetic myopathies, treatment is largely limited to symptomatic management with few disease-modifying options available (123). However, conventional approaches often fail to prevent disease progression or restore muscle function, highlighting the need for targeted therapeutic strategies (122). Recent studies have identified shared pathogenic mechanisms across both genetic and acquired myopathies, particularly involving dysregulated innate immune activation and cellular stress responses (32).

Accumulating evidence indicates that dysregulated innate immune activation contributes to myofiber injury, impaired regeneration, and disease progression in multiple myopathies. Accordingly, current therapeutic approaches aim to modulate distinct but interconnected immune pathways, including inflammasome activation, type I IFN signaling, TNF-α–mediated inflammatory responses, and cytokine receptor signaling pathways that influence muscle inflammation and tissue remodeling.

However, the translational maturity and level of evidence supporting these therapeutic strategies vary substantially among disease categories and therapeutic targets. While some approaches, such as JAK inhibitors in DM, have shown encouraging clinical responses, many other interventions remain limited to preclinical proof-of-concept studies in animal models or early observational studies. In addition, innate immune signaling may play context-dependent roles in both tissue injury and muscle regeneration; therefore, excessive suppression of inflammatory pathways could potentially impair repair-associated processes depending on disease stage and pathological context.

### Targeting NLRP3 inflammasome

The NLRP3 inflammasome plays a critical role in inflammation-associated skeletal muscle wasting, making it an attractive therapeutic target for myopathies characterized by chronic inflammatory stress (**Table 1**). Pharmacological inhibition of NLRP3 has shown protective effects against myofiber damage in several experimental models.

**Table 1:** Representative therapeutic approaches targeting NLRP3 signaling in myopathies.

Therapeutic molecules	Disease/condition	Study type/model	Targets and mechanisms	Results	Translational stage	Reference
Trimetazidine (TMZ)	Muscle atrophy (dexamethasone (DEX)-induced)	DEX-induced mouse atrophy model and DEX-treated C2C12 myotubes	Promotes the phosphorylation of the PI3K/AKT pathway, thereby inhibiting NLRP3/GSDMD.	Protected against skeletal muscle atrophy induced by DEX.	Preclinical	(124)
MCC950	Duchenne muscular dystrophy (DMD)	Human DMD myoblasts and mdx mice	Blocks NLRP3 inflammasome activation by preventing ATP hydrolysis at the NLRP3 NACHT domain.	Reduced inflammation, macrophage infiltration, and oxidative stress in the muscles of mdx mice. Enhanced muscle force and fatigue resistance were observed in mdx mice. Showed anti-inflammatory and anti-pyroptotic effects in human DMD myoblasts.	Preclinical	(125)
Bright blue G (BBG)	Autoimmune myositis	Autoimmune myositis mouse model	Blocks P2X7 receptors, thereby inhibiting pyroptosis.	Reduced muscle NLRP3 expression and serum IL-1β levels in experimental autoimmune myositis mice model.	Preclinical	(126)
Glyburide	Autoimmune myositis	Autoimmune myositis mouse model	Blocks ATP-sensitive potassium (KATP) channels, thereby inhibiting NLRP3 activation.	Reduced muscle NLRP3 expression and serum IL-1β levels in experimental autoimmune myositis mice model.	Preclinical	(126)
Ecklonia (ECE)	Muscle atrophy (dexamethasone (DEX)-induced)	DEX-induced mouse atrophy model	Decreases RAGE and TLR4 expression, resulting in reduced NF-κB signaling, along with suppression of the NLRP3 inflammasome.	Decreased NLRP3 inflammasome/pyroptosis-related proteins along with increased muscle mass, fiber cross-sectional area and grip strength in mice.	Preclinical	(127)
Dieckol	Muscle atrophy (dexamethasone (DEX)-induced)	DEX-induced mouse atrophy model	Decreases RAGE and TLR4 expression, resulting in reduced NF-κB signaling, along with suppression of the NLRP3 inflammasome.	Decreased NLRP3 inflammasome/pyroptosis-related proteins along with increased muscle mass, fiber cross-sectional area and grip strength in mice.	Preclinical	(127)

This table summarizes representative therapeutic strategies targeting NLRP3 inflammasome activation in myopathies, including preclinical experimental studies and emerging translational approaches.

Glucocorticoid-induced muscle atrophy is associated with the activation of NLRP3 inflammasome signaling. Dexamethasone (DEX) induces muscle atrophy accompanied by NLRP3/GSDMD-mediated pyroptosis (124). Trimetazidine (TMZ) mitigates DEX-induced skeletal muscle atrophy by activating the PI3K/AKT pathway, thereby suppressing the NLRP3/GSDMD pathway (124). In a glucocorticoid-induced muscle atrophy model, DEX also enhances HMGB1–TLR4 signaling, promotes NF-κB nuclear translocation, increases the NLRP3 inflammasome–mediated pyroptosis, and upregulates muscle-specific E3 ubiquitin ligases, including MuRF1 and atrogin-1, leading to reduced muscle fiber cross-sectional area and grip strength (127). Treatment with *Ecklonia* (ECE) or dieckol (DK) attenuates the inflammatory and catabolic changes caused by DEX and restores muscle morphology and function in mice (127).

In dystrophin-deficient mdx mice, treatment with MCC950, a selective small-molecule inhibitor of NLRP3, significantly reduces muscle inflammation, macrophage infiltration, and oxidative stress, and improves muscle force generation and resistance to fatigue (125). Consistently, MCC950 exhibits anti-inflammatory and anti-pyroptotic effects in human DMD myoblasts (125). Moreover, MCC950 has been identified as a potential therapeutic target in a broad range of inflammatory conditions, including neurodegenerative diseases (128). In experimental autoimmune myositis (EAM) mice model, inhibition of inflammasome signaling using bright blue G (BBG) or glyburide reduces muscular NLRP3 expression and serum IL-1β levels (126). Moreover, in a streptozotocin-induced mice model of diabetic myopathy, bone morphogenetic protein 7 (BMP-7) is a potential therapeutic agent that attenuates hyperglycemia and suppresses inflammasome activation and pyroptosis in the skeletal muscle (129).

Collectively, these findings suggest that NLRP3 inflammasome inhibition may represent a promising preclinical therapeutic strategy for limiting inflammation-associated muscle damage across multiple experimental models of myopathy. However, further validation is required to determine its disease-specific efficacy, safety, and translational applicability in human myopathies. In addition, although NLRP3 remains the most extensively studied inflammasome in myopathies, other inflammasome-associated pathways may represent future areas of investigation, as current evidence for their pathogenic and therapeutic relevance remains substantially less developed.

### Targeting type I IFN signaling

Type I IFN signaling represents a major pathogenic pathway in DM and related IFN-associated inflammatory myopathies (**Table 2**). Pharmacological inhibition of the JAK–STAT pathway has shown promising effects in early clinical studies aimed at suppressing IFN-driven immune activation. In pilot and open-label clinical studies, treatment with baricitinib, a selective JAK1/2 inhibitor, improves cutaneous disease severity in patients with DM, as assessed by the Cutaneous Dermatomyositis Disease Area and Severity Index (CDASI) and Dermatology Life Quality Index (DLQI) scores, and reduces circulating cytokine levels (130, 132). Similarly, ruxolitinib treatment is associated with improvements in muscle strength, body weight, and cutaneous manifestations in patients with DM, including findings from case-based clinical reports (130, 131). Tofacitinib, a JAK1/3 inhibitor, decreases CDASI activity scores, alleviates pruritus, and improves muscle strength and fatigue in patients with DM without significant adverse effects in small clinical case series (133). Consistently, open-label pilot studies demonstrated that tofacitinib improves overall disease activity according to the 2016 ACR/EULAR myositis response criteria and reduces STAT1 signaling in skin biopsy samples (134).

**Table 2:** Representative therapeutic approaches targeting type I IFN signaling in myopathies.

Therapeutic molecules	Disease/condition	Study type/model	Targets and mechanisms	Results	Translational stage	Reference
Ruxolitinib	Dermatomyositis (DM)	Pilot clinical study in DM patients	JAK1/2 inhibitor	Improved cutaneous disease according to CDASI.	Early clinical	(130)
Ruxolitinib	DM	Case report in refractory DM patients	JAK1/2 inhibitor	Improved muscle strength, body weight, and skin lesions.	Clinical case report	(131)
Baricitinib	DM	Pilot clinical study in DM patients	JAK1/2 inhibitor	Improved cutaneous disease according to CDASI.	Early clinical	(130)
Baricitinib	DM	Prospective open-label study in DM patients	JAK1/2 inhibitor	Improved cutaneous disease according to CDASI, and Dermatology Life Quality Index (DLQI).	Early clinical	(132)
Tofacitinib	DM	Case series in refractory DM patients	JAK1/3 inhibitor	Improved cutaneous disease according to CDASI. Improved strength and fatigue.	Clinical case series	(133)
Tofacitinib	DM	Open-label pilot study in DM patients	JAK1/3 inhibitor	Improved disease activity according to the 2016 ACR-EULAR myositis response criteria. Improved cutaneous disease according to CDASI.	Early clinical	(134)
Sifalimumab	DM and polymyositis (PM) patients	Phase 1b clinical trial in DM and PM patients	Anti–IFN-α monoclonal antibody that suppresses type I IFN signaling	Suppressed the type I IFN gene signature in blood and muscle tissue of DM and PM patients. Targeted neutralization of type I IFN gene signature positively correlates with clinical improvement.	Early clinical	(135)

This table summarizes representative therapeutic approaches targeting type I IFN signaling implicated in inflammatory myopathies, including preclinical, observational, and early clinical studies.

In addition to small-molecule JAK inhibitors, the direct neutralization of type I IFN signaling has shown promise in early clinical trials. Sifalimumab, an anti–IFN-α monoclonal antibody, suppresses the type I IFN gene signature (IFNGS) in both blood and muscle tissue of patients with myositis. In muscle biopsies from patients with DM or PM, sifalimumab inhibits pathways involved in leukocyte extravasation, antigen presentation, and B cell development, and the degree of IFN signature suppression positively correlated with clinical improvement (135).

### Targeting TNF-α signaling

TNF-α contributes to inflammatory muscle damage in a subset of inflammatory myopathies (**Table 3**). Anti–TNF-α therapies have shown variable but potentially beneficial effects across observational and early clinical studies in JDM, DM, and PM. In retrospective studies and clinical case series, treatment with infliximab or adalimumab improves clinical disease activity and muscle involvement in patients with refractory JDM (136, 137). In preliminary clinical studies involving adult DM and PM patients, infliximab reduces circulating pro-inflammatory cytokines, including IL-1β, IL-6, and IFN-γ, improves muscle strength, and decreases muscle fiber necrosis and inflammatory infiltrates (138). Furthermore, randomized placebo-controlled clinical trials demonstrated that infliximab enables a subset of patients with refractory DM and PM to meet the International Myositis Assessment and Clinical Studies (IMACS) definition of improvement (139).

**Table 3:** Representative therapeutic approaches targeting TNF-α signaling in myopathies.

Therapeutic molecules	Disease/condition	Study type/model	Targets and mechanisms	Results	Translational stage	Reference
Infliximab	Juvenile dermatomyositis (JDM)	Retrospective study in JDM patients	Binds to and neutralizes tumor necrosis factor-alpha (TNF-α)	Improved muscle pathology.	Clinical	(136)
Adalimumab	JDM	Retrospective study in JDM patients	Binds to and neutralizes tumor necrosis factor-alpha (TNF-α)	Improved muscle pathology.	Clinical	(136)
Infliximab	JDM	Case series in refractory JDM patients	Binds to and neutralizes tumor necrosis factor-alpha (TNF-α)	Improved muscle pathology.	Clinical case series	(137)
Infliximab	PM (polymyositis) and DM (dermatomyositis)	Preliminary study in DM and PM patients	Binds to and neutralizes tumor necrosis factor-alpha (TNF-α)	Reduced the production of IL-1β, IL-6, and IFN-γ in the blood. Increased skeletal muscle strength, reduced the necrotic muscle fibers and inflammatory infiltrates. Improved myopathic features.	Early clinical	(138)
Infliximab	PM and DM	Randomized placebo-controlled trial in refractory PM and DM patients	Binds to and neutralizes tumor necrosis factor-alpha (TNF-α)	Improved manual muscle strength (MMT) and showed improvement based on IMACS response criteria.	Clinical trial	(139)

This table summarizes representative therapeutic strategies targeting TNF-α signaling pathways implicated in inflammatory myopathies, including observational studies, clinical case series, and controlled clinical trials.

However, therapeutic responses to TNF-α inhibition appear heterogeneous among inflammatory myopathies, and patient selection and disease context may critically influence treatment efficacy. Further controlled clinical studies are required to clarify long-term efficacy, safety, and disease-specific therapeutic applicability.

### Targeting IL receptor signaling

IL-6 signaling has been implicated in muscle inflammation, fibrosis, and impaired muscle regeneration in inflammatory myopathies (**Table 4**). Preclinical studies in dystrophin-deficient models have suggested that IL-6 blockade may improve muscle morphology, reduce dystrophic changes, and promote muscle regeneration (80, 81). In early clinical studies, patients with refractory immune-mediated necrotizing myopathies (IMNMs) respond to treatment with tocilizumab, an anti–IL-6 receptor monoclonal antibody, with responders exhibiting reduced muscle fiber necrosis and decreased fiber size variability (140). Notably, baseline serum IL-6 levels and muscle IL-6 mRNA expression predict therapeutic responsiveness to tocilizumab, highlighting the importance of patient stratification (140).

**Table 4:** Representative therapeutic approaches targeting IL receptor signaling in myopathies.

Therapeutic molecules	Disease/condition	Study type/model	Targets and mechanisms	Results	Translational stage	Reference
MR16–1 antibody	Duchenne muscular dystrophy (DMD)	Preclinical study in dystrophin/utrophin double-knockout (dKO) mice.	Inhibits the IL-6 signaling pathway	Improved muscle fiber diameter and regeneration, and reduced fibrosis in skeletal muscle of dystrophin/utrophin dKO mice.	Preclinical	(81)
Tocilizumab	Immune-mediated necrotizing myopathies (IMNMs)	Open-label pilot study in refractory IMNM patients	Inhibits the IL-6 signaling pathway	Showed clinically meaningful improvement based on the 2016 ACR-EULAR myositis response criteria and decreased muscle fiber necrosis and size variability.	Early clinical	(140)

This table summarizes representative therapeutic strategies targeting IL receptor signaling implicated in myopathies, including preclinical and early clinical studies.

Taken together, these studies highlight the emerging therapeutic relevance of innate immune-associated pathways in myopathies while also emphasizing substantial heterogeneity in mechanistic evidence, translational maturity, and disease-specific applicability across therapeutic strategies. Although several approaches have shown encouraging preclinical or early clinical results, further validation is required to clarify long-term safety, optimal patient selection, disease-stage specificity, and the balance between suppressing pathological inflammation and preserving repair-associated immune responses.

## Concluding remarks

In summary, this review highlights that innate immune activation and regulated myofiber death pathways are implicated across diverse genetic and acquired myopathies, although the strength of mechanistic evidence and the contribution of these pathways vary substantially among disease categories. While the specific upstream triggers and dominant pathological mechanisms differ across myopathies, overlapping innate immune-associated and regulated cell death pathways may contribute to muscle degeneration, impaired regeneration, and tissue remodeling in selected disease contexts. By integrating findings across representative myopathies, this review highlights convergent and disease-specific mechanisms linking innate immune signaling with muscle pathology and discusses their potential relevance for mechanism-based therapeutic strategies.

Future studies should clarify the interactions and potential crosstalk among regulated cell death pathways within myofibers and determine how these mechanisms vary across disease stages, pathological contexts, and myopathy subtypes. In addition, integrating innate immune–targeted strategies with gene-based or other etiology-directed therapies may provide complementary therapeutic benefits; however optimal timing, biomarker-guided patient stratification, and long-term safety considerations remain to be established.

## Glossary

ACE2: angiotensin-converting enzyme 2
AIM2: absent in melanoma 2
CDASI: Cutaneous Dermatomyositis Disease Area and Severity Index
CDM: congenital myotonic dystrophy
CIM: C-protein-induced myositis
CIP/CIM: critical illness polyneuropathy and myopathy
CK: creatine kinase
COVID-19: coronavirus disease 2019
DAMP: damage-associated molecular pattern
DEX: dexamethasone
DM: dermatomyositis
DM1: myotonic dystrophy type 1
DM2: myotonic dystrophy type 2
DMD: Duchenne muscular dystrophy
Fn14: fibroblast growth factor-inducible 14
GSDMD: gasdermin D
GSDME: gasdermin E
HMGB1: high-mobility group box 1
HSP70: heat shock protein 70
IBM: inclusion body myositis
IFN: interferon
IFNAR: interferon-α/β
IIM: idiopathic inflammatory myopathy
IL: interleukin
IMNM: immune-mediated necrotizing myopathy
ISG: interferon-stimulated gene
JDM: juvenile dermatomyositis
MLKL: mixed-lineage kinase domain-like protein
MuRF1: muscle RING finger 1
MxA: myxovirus resistance protein A
MyD88: myeloid differentiation primary response 88
NF-κB: nuclear factor kappa B
NLRP3: NOD-like receptor family pyrin domain-containing 3
PAMP: pathogen-associated molecular pattern
PBMC: peripheral blood mononuclear cell
PM: polymyositis
PRR: pattern-recognition receptor
RIPK1: receptor-interacting protein kinase 1
RIPK3: receptor-interacting protein kinase 3
SAA1: serum amyloid A1
SARS-CoV-2: severe acute respiratory syndrome coronavirus 2
STAT3: signal transducer and activator of transcription 3
TLR: Toll-like receptor
TMZ: trimetazidine
TNF-α: tumor necrosis factor-alpha
TNFR1: tumor necrosis factor receptor 1
TWEAK: TNF-like weak inducer of apoptosis
ZBP1: Z-DNA binding protein 1
